# Solid peripheral tumor leads to systemic inflammation, astrocyte activation and signs of behavioral despair in mice

**DOI:** 10.1371/journal.pone.0207241

**Published:** 2018-11-15

**Authors:** Melanie Demers, Georgette L. Suidan, Nick Andrews, Kimberly Martinod, Jessica E. Cabral, Denisa D. Wagner

**Affiliations:** 1 Program in Cellular and Molecular Medicine, Boston Children’s Hospital, Boston, Massachusetts, United States of America; 2 Department of Pediatrics, Harvard Medical School, Boston, Massachusetts, United States of America; 3 Kirby Neurobiology Center, Boston, Children’s Hospital and Department of Neurobiology, Harvard Medical School, Boston, Massachusetts, United States of America; 4 Graduate Program in Immunology, Division of Medical Sciences, Harvard Medical School, Boston, Massachusetts, United States of America; Rutgers University, UNITED STATES

## Abstract

Prevalence of depression is higher in patients with cancer than in the general population. Sustained systemic inflammation has been associated with depressive behavior and it has been reported that depressed patients commonly display alterations in their immune system. We previously showed that cancer in mice induces a systemic environment that promotes neutrophil activation and leukocytosis. We thus hypothesized that the peripheral systemic response to a solid tumor leads to endothelial activation, which may promote inflammatory changes in the brain with behavioral consequences. Using the Lewis lung carcinoma (LLC) model, we show that tumor growth induces a progressive increase in peripheral inflammation as observed by elevated interleukin-6 (IL-6). In behavioral studies, tumor-bearing mice showed no sign of motor, coordination or short term working memory deficits as assessed by rotarod, balance-beam, and novel object recognition tests. However, there was an impairment in the grip strength test and interestingly, an anxious and despair-like phenotype in the elevated plus-maze, and tail suspension tests, respectively. Immunostaining of perfused brains revealed fibrin accumulation in the vasculature with some leakage into the parenchyma, a process known to activate endothelial cells. Taken together, our results suggest that the inflamed and prothrombotic systemic environment created by the growth of a peripherally-located solid tumor induces endothelial activation, accumulation of fibrin in the brain and astrocyte activation, perhaps leading to depressive-like behavior.

## Introduction

Prevalence of depression is higher in patients with cancer than in the general population and is associated with increased morbidity. Hearing distressful news such as a cancer diagnosis can factor in to a depressive state. However, it has also been postulated that activation of pro-inflammatory cytokines as well as alterations of the hemostatic system can contribute to depression (reviewed in [1]). Peripheral inflammation has been shown to induce astrocyte activation in the hippocampal region of the brain [2,3], a highly plastic and stress-sensitive region that plays a central role in depressive-like behavior [4].

Astrocytes make up the most mass in the brain and are the main support cell to neurons as well as the endothelium, providing a liaison between the two. They are vital for structural normalcy in the brain and play a hefty role in maintenance of homeostasis in the CNS. Astrocytes are also implicated in development and maintenance of the blood-brain barrier. Forming prominent borders that line all interfaces between CNS and blood vessels, astrocytes can display anti-inflammatory barrier functions preventing neurotoxic inflammation or a pro-inflammatory phenotype resulting in a cytotoxic profile [5].

Systemic inflammation and vascular dysfunctions are often observed in cancer patients and mouse models of cancer [6,7]. Our group documented in mice that several types of primary tumors induce a systemic inflammatory environment, which lead to leukocytosis, neutrophil and endothelial activation, and ultimately cancer-associated thrombosis [8].

Here we show, following the subcutaneous injection of Lewis Lung carcinoma (LLC) cells which induces a mild systemic inflammatory environment [8,9], that the mice with a tumor also develop progressive leukocytosis and endothelial activation with accumulation of fibrinogen. This systemic inflammation was associated with an anxious and despair-like phenotype. Additional behavioral studies indicated that tumor-bearing mice showed no sign of motor coordination or short term working memory deficits. Interestingly, the systemic activation induced by the peripheral solid tumor was accompanied by an increase in astrocyte activation and fibrinogen deposition in the brain vasculature. Together, our results suggest that the induction of astrocyte activation through a systemic inflammatory/procoagulant state could contribute to the depressive-like phenotype associated with cancer.

## Material and methods

### Cell lines and reagents

The LLC cell line was purchased from ATCC and maintained in high glucose Dulbecco’s Modified Eagle’s Medium (DMEM) + L-GlutaMAX-1 [supplemented with 10% (v/v) FCS, 10 mM HEPES buffer]. LLC cells originated from C57BL/6 mice and were characterized by ATCC according to the cell line authentification testing (growth curve analysis; mycoplasma, bacteria, and fungi contamination; DNA profiling; and species confirmation) and were used within 6 months after resuscitation. All cell culture products were purchased from Life Technologies/Invitrogen.

### Animals

All animal procedures were performed using adult, female (6 to 10 weeks), C57BL/6J mice (The Jackson Laboratory) and approved by the Institutional Animal Care and Use Committee of Boston Children’s Hospital (Protocol number: 11-04-1941, 14-04-2633). Animals were group-housed, at a maximum of five per cage, on a 12-hour light/dark cycle. Food and water were provided ad libitum. Number of animals used in this study was based on previous work done with this model system [8].

### Induction of solid tumors

LLC (5 x 10^5^) cells were inoculated subcutaneously in the right flank. The tumor volume was calculated using the formula *V* = *lw*^*2*^ x 0.4 where *l* is the length and *w* the width [10,11,12]. Mice were monitored twice weekly by study participants at which time tumors were measured using calipers. Mice were euthanized prior to study endpoints if tumors were ulcerated or if the animals were moribund (obvious lack of grooming, extreme weight loss, signs of dehydration) or tumors were larger than 2500mm^3^. In this study, no animals were euthanized prior to study endpoints. Analgesic were not administered at any point during the study until euthanization. Blood was collected using EDTA-coated capillaries and analyzed with a Hemavet hematology analyzer (Drew Scientific). At sacrifice, blood was collected into 3.2% sodium citrate, centrifuged at 6000 rpm for plasma collection and centrifuged again at 12000 rpm to remove any contaminating cells. For brain collection, animals were perfused with 15ml of PBS under ketamine and xylazine anesthesia.

### Plasma analyses

Plasma IL-6 and fibrinogen were assessed using Mouse IL-6 ELISA (Abcam) and Mouse fibrinogen ELISA (Genway Biotech), following manufacturer’s instructions. The VWF ELISA was performed as previously described [13] using the level of VWF in pooled plasma of 20 C57BL/6J WT mice as a reference standard (nmp: normal mouse plasma).

### Behavioral tests

#### General information

We used the C57BL/6 stratin of mice because they are syngeneic to the cancer cells. The behavioral experiments were performed at the Kirby Biology Center which is part of the Dept of Neurobiology at Harvard Medical School. The Neurodevelopmental Behavior Core has a housing facility within the footprint to minimize transfer stress to the testing rooms. Mice were left for at least 30 min to acclimatize to the test room before experiments were begun. The testing was performed in rooms with low light (30 lux) to reduce stress. The person performing the test left the room during each test to minimize any influence on the behavior of the mouse during the trial.

#### Novel object recognition

Animals were allowed to adapt to the chamber for 5 minutes without objects prior to testing. The test consisted of 2 phases: Sample Phase, where animals were allowed to explore an arena containing two identical objects until they had investigated both objects for a total period of 20 seconds; Recall Phase, where animals were allowed 5 minutes to explore the arena and two objects, one of which was the same as in the sample phase and one of which was novel. The recall phase occurred 10 minutes after the sample phase. The test started by the animal being placed into the arena at the far side against the wall and opposite to where the objects were located. Behavior was recorded remotely by a camera and the amount of time in seconds spent sniffing the objects was analysed using Ethovision XT v11.5. Only time spent with the nose in contact with an object was considered as investigation of that object. Objects in both phases of the test were alternated across trials to control for possible side bias. Objects and arena were cleaned thoroughly between trials with 10% bleach solution. Time spent with the novel object was divided by the time spent with the familiar object to obtain a novelty ratio for each mouse.

#### Elevated plus maze

The elevated plus maze consisted of a device with two “open” arms that had no walls and two “closed” arms (30x5cm) with walls arranged to create a “plus” shape. The area where the arms cross in the center formed the decision zone. The entire apparatus was raised 85 cm above the floor (Lafayette Instruments). Mice were placed on the center platform of the maze, facing a closed arm, and allowed to explore the apparatus for 5 minutes. The maze was cleaned between subjects with 10% bleach and dried. Behavior was recorded remotely by a camera and the total time spent in the open, center, and closed compartments was analysed using (Noldus Ethovision XT v11.5). Time spent on the open arm was divided by the time in the closed arms to calculate % time on the open arms for each mouse.

#### Tail suspension

Mice were suspended by the tail (taped onto a suspension hook so that the animal hung with its tail in a straight line) for a test duration of 6 minutes about 30–50 cm above the surface of a table covered with soft padding material. The duration of immobility was scored during the last 4 minutes of the test for each mouse using ethovision XT v11.5.

### Motor and balance tests

#### Beam walk

The beam was a narrow metal rod (1m long and 1cm wide) 50 cm above the bench surface with a black plastic box at one end in which cage mates of the test mouse were placed to encourage the test mouse to walk across the beam. A camera was located 90 degrees, perpendicular to the beam such that the whole length of the beam could be captured. The mice were trained the day before the first test to traverse the beam in both directions. The following day the test mouse was placed at one end of the beam and allowed to freely traverse along the beam in one direction. Having completed the first trip the mouse was then tested in the opposite direction. This allowed both sides of the animal to be assessed for foot slips in post-hoc review of the video recordings. Time taken to traverse the beam, total footsteps and number of foot slips for each mouse were scored by an observer blind to group.

#### Rotarod

Animals were placed onto the device on the day prior to test and trained to remain on the rotating rod for a period 5 min. The rod rotated at a fixed speed of 4 rpm during training. If a mouse fell off the rod, it was placed back onto the device without interruption of the rotations. The following day mice were placed on the rod at 4 rpm for ten seconds to adjust to the rod speed after which the rod accelerated at 0.1rpm/sec. Each mouse completed five trials on the test day with a minimum of five minutes rest between each trial. The mean time spent on the rod was calculated from the 5 trials for each mouse.

#### Grip strength

Motor function and coordination were evaluated by the grip test as modified from Moran et al., PNAS 1995. For this test, a mouse was placed in the center of a semi-flexible wooden rod (3 mm in diameter, 40 cm long) fixed between two vertical supports, elevated 40 cm from the benchtop. Mice were rated within 30 seconds with the following scoring system: 0, mouse fails to grab onto the rod and/or falls off; 1, mouse hangs onto the rod by one or both forepaws; 2, as for 1, and attempts to climb onto the rod; 3, hangs onto rod by one or both forepaws plus one or both hindpaws; 4, mouse hangs on with forepaws and hindpaws plus the tail wrapped around rod; 5, mouse escapes to the supports.

### Immunostaining

Brains were harvested from perfused animals and snap frozen on dry ice. Cryosections were fixed with zinc fixative and stained with a sheep anti-fibrinogen antibody (ABD Biologicals), and incubated with anti-sheep Alexa-488 or Alexa-555 (Invitrogen) as secondary antibodies for fibrinogen, and Cy3-conjugated GFAP (Sigma #9205) or FITC-conjugated lectin from Lycopersicon esculentum Sigma (L0401). Sections were counterstained with Hoechst-33342 to visualize all nuclei, mounted with Fluoro-gel (Electron Microscopy Sciences) and observed under an epifluorescent Axiovert microscope (Zeiss). All images were taken using the same exposure settings per magnification.

### BBB permeability

Brains were harvested from perfused animals and snap frozen on dry ice. Thin sections throughout the whole brains were collected and lysed by sonication in RIPA buffer (Sigma #R0278) containing protease and phosphatase inhibitors (Thermo Scientific # 88669). The lysate was assessed for fibrin(ogen) using a mouse fibrinogen ELISA (GenWay #BB0BA2). Evan’s blue dye assay was performed on a separate set of animals and was assessed as previously described [14].

### Western blotting

Thin section lysates used for fibrinogen ELISA were also used for western blotting experiments. Samples were normalized for protein (BCA kit #) and added to Laemmli buffer (Bio-rad) containing 2-marcaptoethanol and boiled for 5 minutes. After cooling, samples were loaded onto 4–20% SDS-PAGE gels and transferred onto Immobilon PVDF membranes. Blots were probed for GFAP and GAPDH (Fitzgerald industries International). Western blots were quantified using Image J64 software (National Institutes of Health). For quantification, values for GFAP were normalized to GAPDH (protein loading control).

### Statistical analysis

Data are represented as mean ± SEM and were analyzed by One-way ANOVA followed by Bonferroni’s post-hoc analyses. All p-values were considered significant below 0.05.

## Results

### Solid LLC tumor induces systemic inflammation and endothelial activation

**Fig 1:**
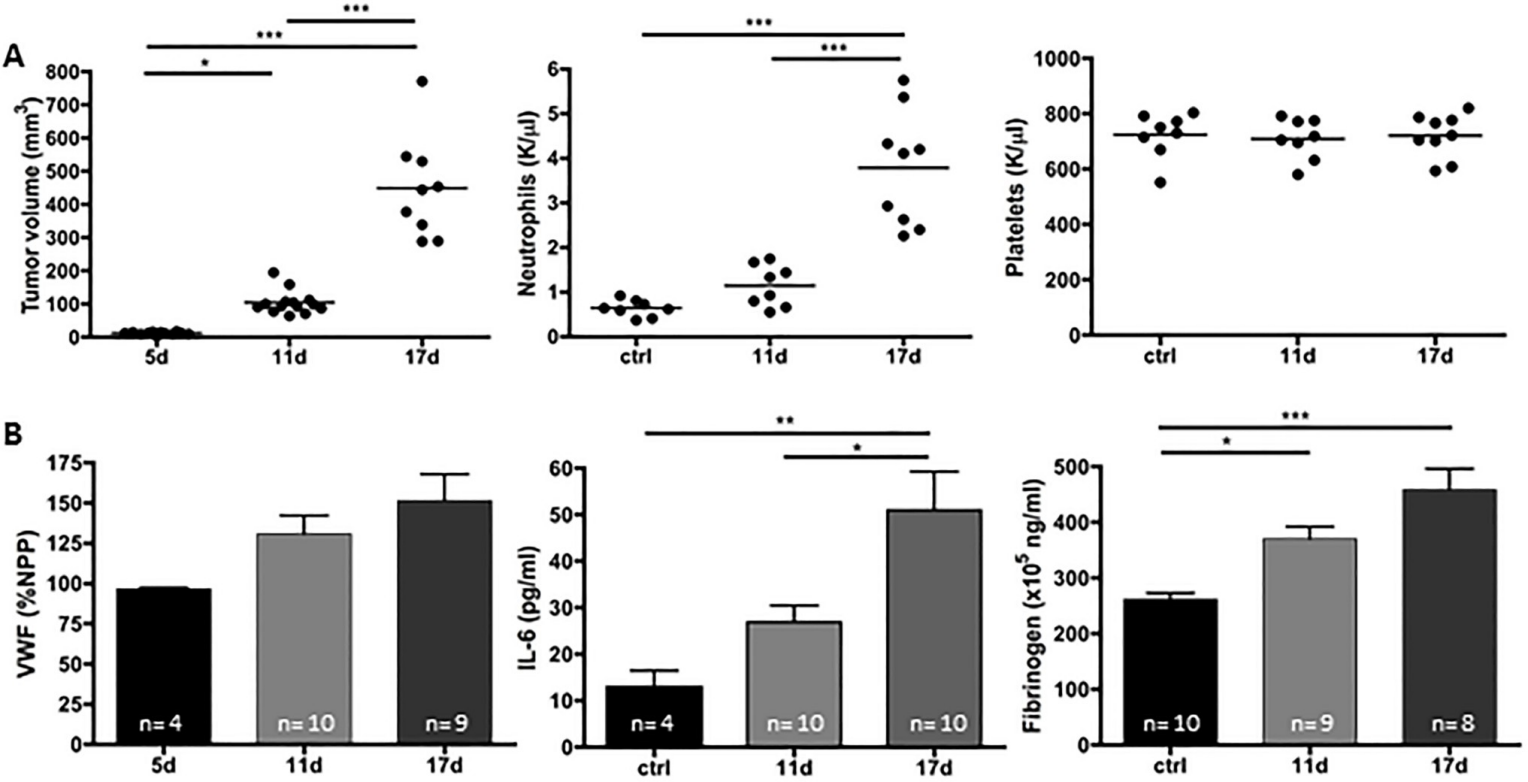
Subcutaneous proliferation of LLC cells induces systemic inflammation associated with neutrophilia and fibrinogen elevation. LLC cells were injected in the right flank and tumors were allowed to grow. (A) Tumor growth and blood count were evaluated at day 11 and 17 post-implantation. An increase in neutrophil but not platelet count was observed with tumor growth. (B) Plasma analysis revealed increase in interleukin-6, and fibrinogen in tumor-bearing mice compared to controls. Elevation of von Willebrand Factor did not reach statistical significance but likely shows an increase in endothelial activation (n = 4–10; *p<0.05, **p<0.01, ***p<0.005). Statistical analyses were performed using One-way ANOVA with Bonferroni’s post hoc analysis.

Peripheral solid tumors often induce a systemic inflammatory state that correlate with tumor growth in mouse models and cancer patients [15,16]. To further evaluate the development of the inflammatory state during peripheral tumor growth, we subcutaneously injected mice with LLC tumor cells and allowed tumors to grow. At days 11 and 17 post-implantation, when no signs of distant metastasis can be observed (as assessed by histological Hematoxylin and Eosin staining-data not shown), blood cell counts and plasma analyses were performed. A progressive increase in blood neutrophil counts but not platelets was observed as the tumor grew (Fig 1A). Plasma IL-6 also showed a progressive increase as there was a significant increase between day 11 and day 17. Plasma VWF and fibrinogen levels showed a trend towards an increase although there was not a significant increase between days 11 and 17 Together, these data suggest a gradual elevation in systemic inflammation and endothelial activation (Fig 1B).

### LLC-tumor-bearing mice show normal motor abilities but demonstrate an anxious and depressive-like phenotype

**Fig 2:**
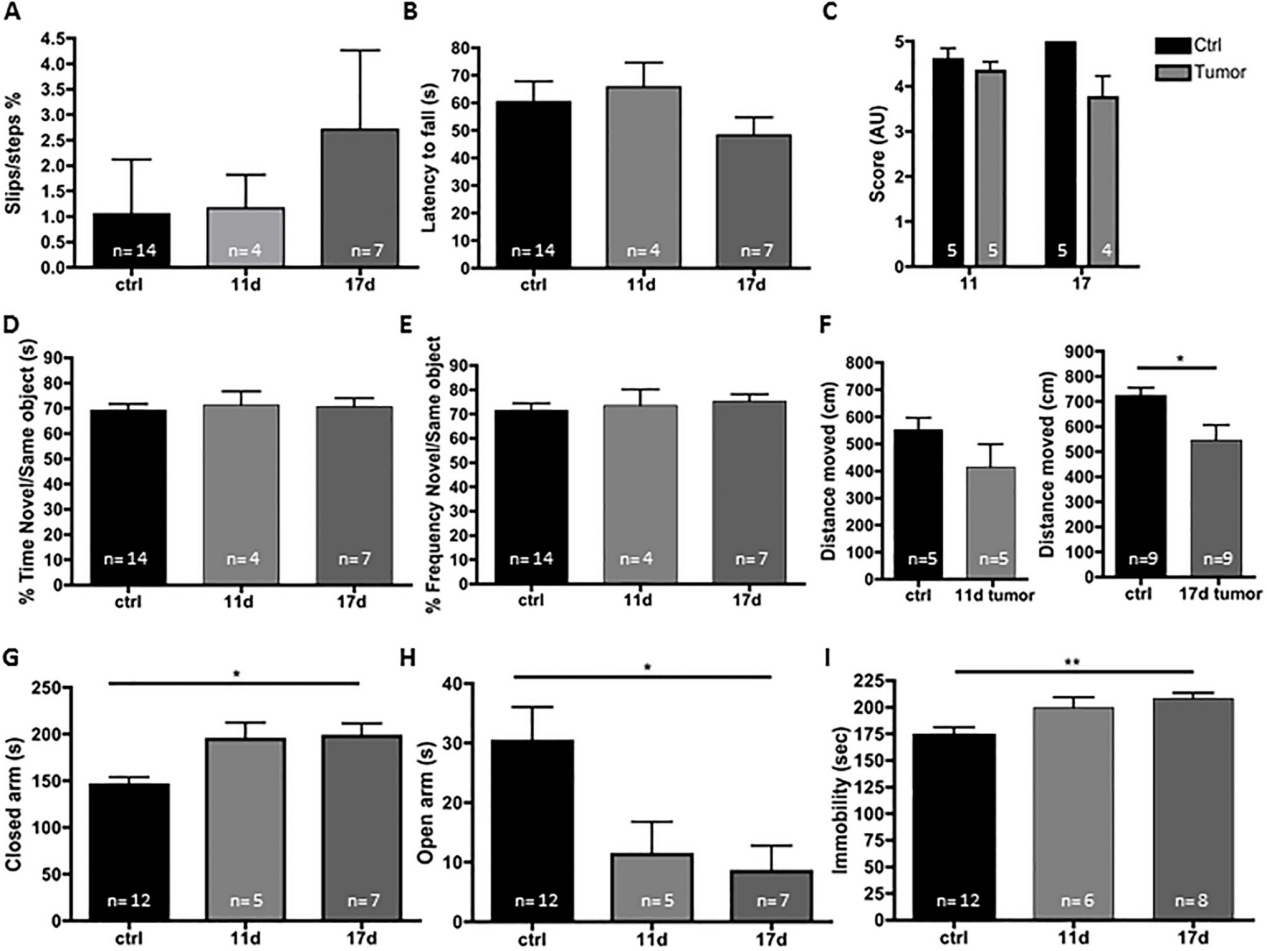
Tumor-bearing mice show a more anxious and depressive-like phenotype without signs of motor, coordination, or short-term working memory deficit. LLC cells were injected in the right flank and tumors were allowed to grow. At day 11 and 17 post-transplantation, behavioral studies demonstrated that tumor-bearing mice are more anxious and show signs of despair. The presence of a tumor was not associated with deficit in motor/coordination as revealed by the similar numbers of slips in the beam walk test (A) and latency to fall from the rotarod (B). However, the grip strength test trended towards a lower score for the tumor-bearing mice 17 days post-implantation (C). No deficits in short term working memory was observed with the novel object recognition test (D-E) as the tumor- bearing mice showed a similar interest for the novel object as control mice. The total distance moved was significantly less in tumor-bearing mice 17 days post-implantation (F). Tumor-bearing mice spent more time in closed arms areas and less in the open arms areas of the elevated-plus maze test (G-H). The tail suspension test (I) showed that mice with tumors were longer immobile. (n = 4–14; *p<0.05, **p<0.01). One-way ANOVA was used to calculate significant differences between groups.

As cancer patients and patients with chronic inflammation have been reported to suffer from depression and anxiety, we performed a battery of behavioral tests on control and tumor-bearing mice on days 11 and 17 after implantation. Tumor-bearing mice demonstrated normal motor abilities as revealed by the same number of slips in the beam walk test (Fig 2A) and the same latency to fall from the rotarod as controls (Fig 2B) with the exception of the grip test (Fig 2C), in which the tumor-bearing mice had a tendency to perform worse than controls by day 17 after LLC inoculation. A weakness in the grip test, which is typically used to measure forelimb motor function, has also previously been associated with depression [17] as it could show the unwillingness of the mouse to persevere. Assessment of exploration of a novel object was similar in tumor-bearing mice and control animals although the distance moved was significantly less at day 17 post-implantation. This may be a sign of diminished overall health of the animal (Fig 2D–2F). Interestingly, tumor-bearing mice spent significantly more time in the closed arm (Fig 2G) and significantly less time in the open arm (Fig 2H) of the elevated plus maze (EPM) indicating that these mice show signs of increased anxiety. Tumor-bearing mice traveled the same distance while in the EPM as control mice, further indicating that they have normal motor capability (data not shown). In the tail suspension test (Fig 2I), tumor-bearing mice at days 11 and 17 demonstrated increased immobility duration when compared to control mice, indicating a “depressive-like” phenotype. These data support previous findings showing that peripheral cancers can lead to a depression-like phenotype [18,19].

### Peripheral solid LLC tumor induces astrocyte activation

**Fig 3:**
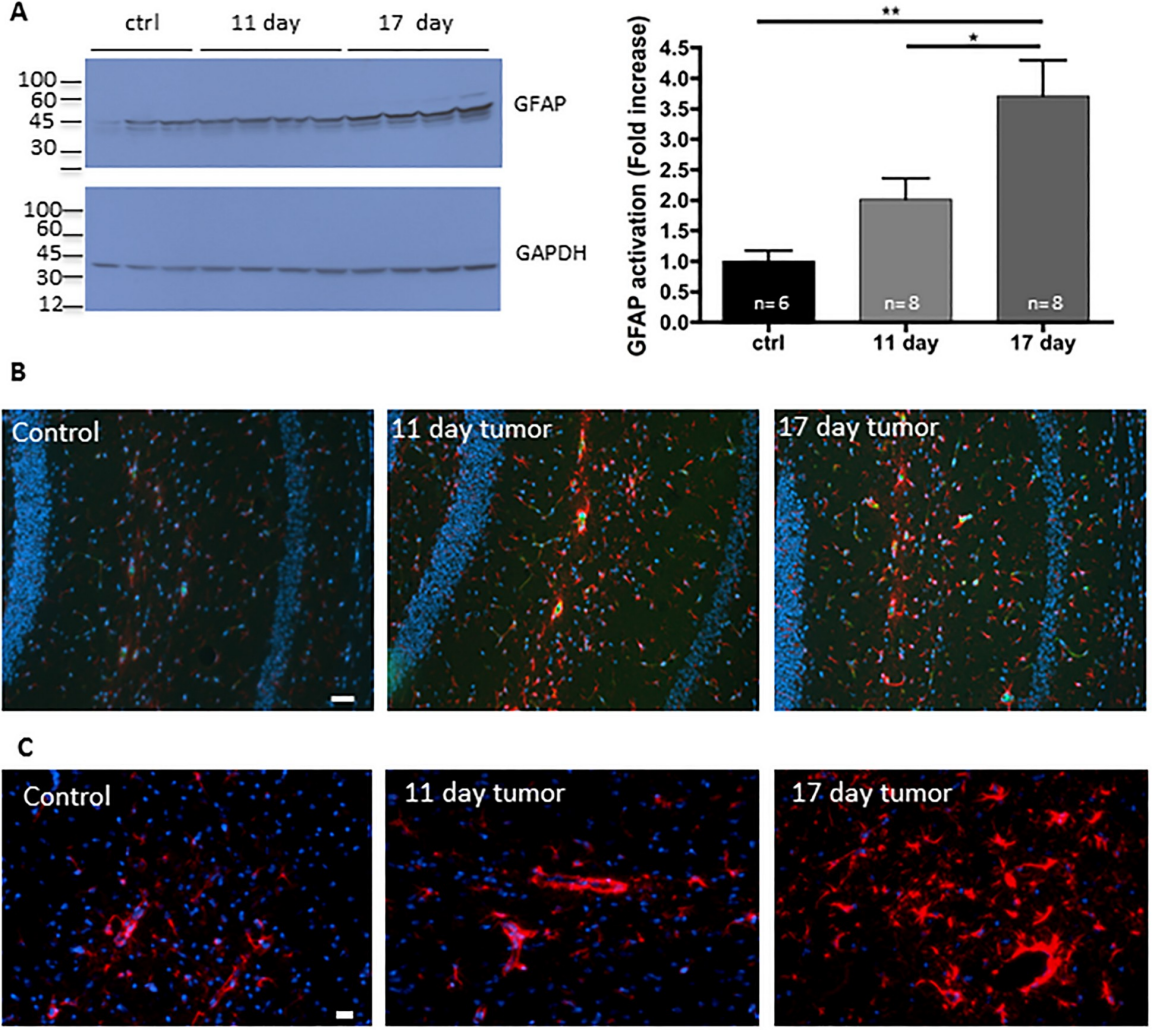
Increase in astrocyte activation in the brain of tumor-bearing mice. LLC cells were injected in the right flank and tumors were allowed to grow. At day 11 and 17 post-transplantation, mice were perfused and the brains were collected. (A) Brain homogenates were evaluated for GFAP, a marker of astrocyte activation, by western blot and quantified. (n = 6–8; *p<0.05, ** p<0.01). (B) Fibrin(ogen) (green) and GFAP (red) immunostaining of the brains revealed an increase in astrocyte activation and accumulation of fibrin(ogen) in blood vessels. Bar 50 μm. (C) Higher magnification showing astrocyte activation by GFAP immunostaining of the hippocampus. Bar 20 μm. Slides were counterstained with Hoechst 33342 for DNA (blue).

Emerging studies associate depression-like symptom with modulation of the immune system [20]. Indeed, recently, several models of systemic inflammation have been shown to cause astroglyosis, the increased proliferation of astrocytes (Reviewed in [21]). To determine whether the systemic inflammation and endothelial activation in tumor-bearing mice lead to astrocyte activation, we assessed levels of glial fibrillary acidic protein (GFAP), an intermediate filament protein used as astrocyte activation marker, in the whole cerebrum lysates from these mice. GFAP protein levels in these homogenates were significantly increased between control and day 11 and were significantly elevated between days 11 and 17 (Fig 3A) indicating wide-spread activation of astrocytes. GFAP immunostaining specifically in the hippocampus also showed a marked intensity increase by day 11 after tumor cell implantation that remained intense at day 17 (Fig 3B and 3C) further confirming astrocyte activation. If activation only occurred in the hippocampus, it would be unlikely that we would see a change by western blot of the whole cerebrum.

### Fibrin(ogen) accumulates in cerebral vessels of tumor-bearing mice

**Fig 4:**
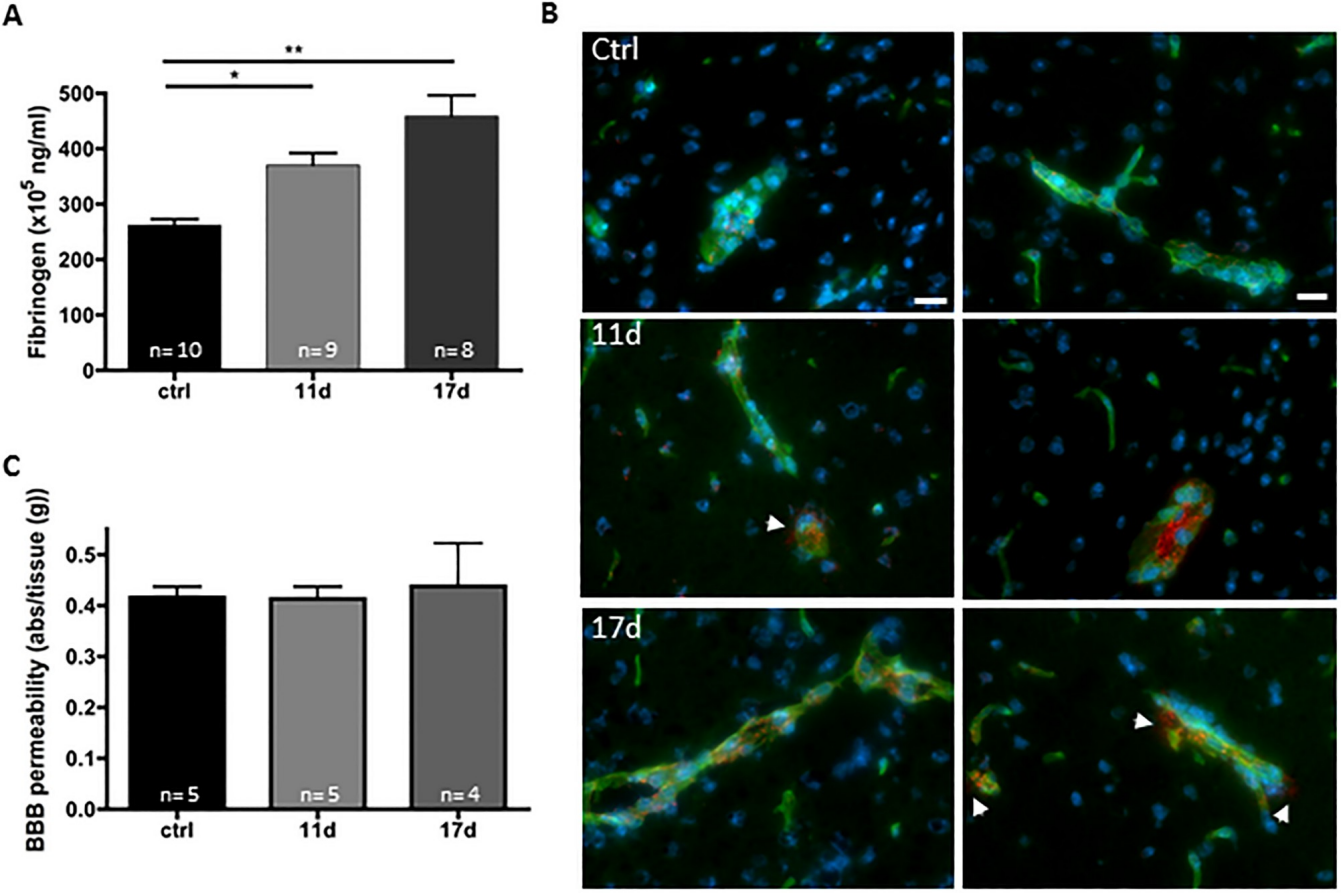
Vascular accumulation of fibrin(ogen) in the brain of tumor-bearing mice. LLC cells were injected in the right flank and tumors were allowed to grow. At day 11 and 17 post- transplantation, mice were perfused and the brains were collected. (A) Brain homogenates were evaluated for fibrin(ogen) levels by ELISA and showed an increase with time after implantation. (n = 4–5, * p<0.05, ** p<0.01). (B) Immunostaining of the brains revealed that fibrin(ogen) (red) mostly accumulates at the vessel wall (lectin; green) in tumor-bearing mice. Minor leakage of fibrin(ogen) into parenchyma (arrows) can also be observed. Bar 20 μm. Slides were counterstained with Hoechst 33342 for DNA (blue).

Since astrocytes play a critical role in maintenance and formation of the blood brain barrier (BBB) phenotype (reviewed in [22]) we evaluated BBB integrity. To determine the status of BBB integrity in tumor-bearing mice, we collected perfused brains from control mice and tumor-bearing mice 11 and 17 days after inoculation with LLC cells. By ELISA, we determined that brain fibrin(ogen) levels were significantly increased compared to controls by day 11 and were further elevated at day 17 (Fig 4A). As fibrin can accumulate on the activated endothelial wall and not be removed by perfusion, we sought to determine localization of this protein in brains of tumor-bearing mice (Fig 4B). Interestingly, we found that almost all fibrin(ogen) was localized to the cerebral vessels with very little appearing to be extravascular. Fibrin formation on endothelium causes endothelial activation, manifested by endothelial granule release of VWF [23]. BBB integrity was also assessed by determining leakage of Evan’s blue dye bound to albumin in control and tumor-bearing mice (Fig 4C). Only one animal in the 17 day group showed significant increase in leakage of plasma protein into the brain further suggesting that the BBB was intact in the large majority of the tumor-bearing mice. Taken together, these results suggest that the observed increase in astrocyte activation more likely results from the accumulation of fibrin on the vessel wall and endothelial stimulation than from a major breakdown of the BBB.

## Discussion

Anxiety and depression are common among patients with cancer and clinical evidence now suggests a causative effect of cancer on the initiation of depressive symptoms, as the psychological behavior is exhibited before the diagnosis of cancer [24]. Inflammation is starting to be recognized as a potential physiological contributor to depression [25]. Here, we showed that LLC tumor-bearing mice display a systemic inflammatory state and a despair-like phenotype. The depressive-like symptoms were observed when accumulation of fibrin/ogen was present in the brain vasculature and was associated with astrocyte activation. Taken together our results suggest that astrocyte activation occurs in systemic inflammatory/procoagulant state caused by cancer and could potentially play a role in the behavioral despair phenotype observed in the tumor-bearing mice and possibly cancer patients.

Depression-like symptoms in tumor-bearing mice have been observed in mouse models of breast, ovarian and colon carcinoma [19,26,27,28,29]. Increased anxiety-like behavior has also been shown in the marble-burying test in rats inoculated with breast cancer [29]. We report here that LLC tumor-bearing mice have a depression-like phenotype and increased anxiety. Previous studies have suggested that depressive-like behavior is related to the systemic elevation of cytokines, such as IL-6 and IL-1B [26,27,28,29]. Our results demonstrate that in addition to the release of peripheral cytokines, the tumor burden induces endothelial activation and a procoagulant state that leads to the accumulation of fibrin within the brain vasculature and importantly astrocyte activation. Although no obvious breakdown of the BBB was observed, it is possible that the accumulation of fibrin on the vessels locally loosens the tight junctions and induces permeability leading to the release of plasma with cytokines in the brain. Indeed, fibrin(ogen) has also been shown to reduce the expression level of tight junction proteins and to induce endothelial cell permeability [30,31] resulting in astrocyte activation. Cytokines do not normally cross the BBB but could enter the CNS at sites where the BBB has been compromised [26]. This is also consistent with our findings of a slight extravascular deposition of fibrin surrounding the vessels. Fibrin deposition on endothelium can also induce a reduction in blood flow which is known to lead to cognitive changes [32,33]. Furthermore, chronic cerebral hypoperfusion has been shown to activate astrocytes in mice [34]. Release of VWF upon fibrin deposition [23] also leads to the accumulation of platelets, monocytes and neutrophils at the vessel wall. P-selectin dependent monocyte rolling on the vessel wall without extravasation induced activation of microglia and sickness behavior in a bile duct ligation model [35]. We previously reported that cancer (including LLC) stimulates neutrophil extracellular trap (NET) formation and NET release may increase further the procoagulant state [8]. Neutrophils/NETs have also been implicated in nervous system diseases associated with increased astrocyte activation such as stroke and Alzheimer’s disease [36,37,38]. Further studies are underway to determine the role of NETs in initiation of neuroinflammation and depressive-like behavior in cancer models.

Although astrocyte activation has been widely related to systemic inflammation, to our knowledge, this is the first report of peripheral tumor burden leading to astrocyte activation. Our results show a progressive increase in astrocyte activation as the inflammatory/procoagulant state of tumor-bearing mice progresses. Studies showing astrocyte activation in mouse models widely use systemic injection of bacterial lipopolysaccharides (LPS). In this setting, activation of astrocytes is maintained for 24h with a peak at 6h which follow the released of inflammatory cytokines [39]. Moreover, repeated injection of LPS showed a rapid increase followed by a decrease in activation of astrocytes two days after the initiation of the treatment and was thought to be related to the immune tolerance induced by LPS [39]. Although the peripheral injection of LPS also induces an inflammatory/procoagulant state, the astrocyte activation induced in our model follows a progression that is similar to tumor growth. In our model, we see a gradual and sustained increase in peripheral cytokines and procoagulant activity, fibrinogen accumulation at the cerebrovascular wall and astrocyte activation (central inflammation). This is quite different from the repeated variation in the levels of cytokines induced by LPS injections. Thus, this model is more representative of a chronic inflammatory condition leading to depressive-like symptoms.

## Conclusion

Our study indicates that the peripheral inflammation/procoagulant state produced by the tumor burden induces fibrin accumulation at the vessel wall in the brain. Astrocyte activation is also occurring, which could be the link to anxiety and signs of behavioral despair. Thus, our data suggest that anti-inflammatory treatment of the chronic inflammation observed in cancer patients might also treat or prevent depression. Indeed, ibuprofen has been shown to reduce the depressive behavior of tumor-bearing mice [40]. Interestingly in an independent study from 2013 [41], we have reported that peripheral serotonin, which is stored in platelets and released upon activation, promotes the recruitment of neutrophils at sites of inflammation. Blocking serotonin uptake with fluoxetine, the gold-standard drug used to treat depression, reduced drastically peripheral serotonin level in the mice as well as endothelial activation and leukocyte recruitment to the vessel wall, diminishing inflammation. Our work suggests that therapies targeting general inflammation and the resulting astrocyte activation, in cancer patients may alleviate anxiety and depression.

## Supporting information

S1 Checklist (PDF)
